# A revision of the Neotropical caddisfly genus *Ascotrichia* Flint, 1983 (Trichoptera, Hydroptilidae)

**DOI:** 10.7717/peerj.7560

**Published:** 2019-08-20

**Authors:** Robin E. Thomson

**Affiliations:** Department of Entomology, University of Minnesota—Twin Cities Campus, Saint Paul, MN, USA

**Keywords:** Trichoptera, Caddisflies, *Ascotrichia*, New species, Hydroptilidae, Neotropical

## Abstract

A revision of the microcaddisfly genus *Ascotrichia* (Trichoptera, Hydroptilidae) is provided, including a generic diagnosis, illustrations, and descriptions of males. This genus is endemic to the Neotropical region and has been recorded from countries in northern South America. Adults of the genus are notable within the family for the contrasting black and green hairs on the forewings. A total of six species are treated, three described as new: *Ascotrichia adirecta* sp. n. (Brazil), *A. hystricosa* sp. n. (Brazil), and *A. simoma* sp. n. (Brazil).

## Introduction

The genus *Ascotrichia* Flint, 1983 belongs to the family Hydroptilidae, the micro or case-making caddisflies. The genus was originally established for two species: *Ascotrichia frontalis* Flint, 1983, the type species, and *A. surinamensis* (Flint), 1974, transferred from the genus *Betrichia* Mosely, 1939. *Ascotrichia spangleri* Oláh and Flint was described in 2012, bringing the total number of species contained within the genus to three (Holzenthal & Calor, 2017). The genus is endemic to the New World and its distribution includes northern and eastern South America (Table 1).

**Table 1:** *Ascotrichia* species and distributions (Trichoptera, Hydroptilidae).

*Ascotrichia* species	Distribution
*adirecta* sp. n.	Brazil
*frontalis* Flint, 1983	Brazil, Paraguay, Uruguay
*hystricosa* sp. n.	Brazil
*simoma* sp. n.	Brazil
*spangleri* Oláh & Flint, 2012	Venezuela
*surinamensis* (Flint, 1974)	French Guiana, Guyana, Suriname

In the original description, Flint (1983) described a head with large posterior warts and a large setae-filled pocket anteriorly, a forewing with a basally enlarged R vein bearing a row of erect setae, and a 1, 3, 4 tibial spur formula. Structures of the maxillary and labial palpi were not described; no illustrations of the wings, head, antennae, or legs were provided. The description of the male genitalia referred to posteromesal points on sterna VI and VII, a broad sternum VIII with a posterolateral process, and a phallus with a midlength complex. More detailed features of the male genitalia and a forewing length of 4.5–5 mm were given in the species description of *A. frontalis*. Male genitalia features included a V-shaped ventromesal excision in the posterior margin of sternum VIII; a shallowly bifid, laterally flared apex to the subgenital plate; a single, slender, curved process arising ventrolaterally from segment IX; and mesally fused inferior appendages. Illustrations of the male genitalia were included in the description of the type species (Flint, 1983, figs. 111–114). Flint suggested that *Ascotrichia* may be most closely related to the genus *Abtrichia* Mosely, 1939, and could be separated by the lack of extremely modified basal antennal segments that are present in *Abtrichia* species.

**Figure 1:**
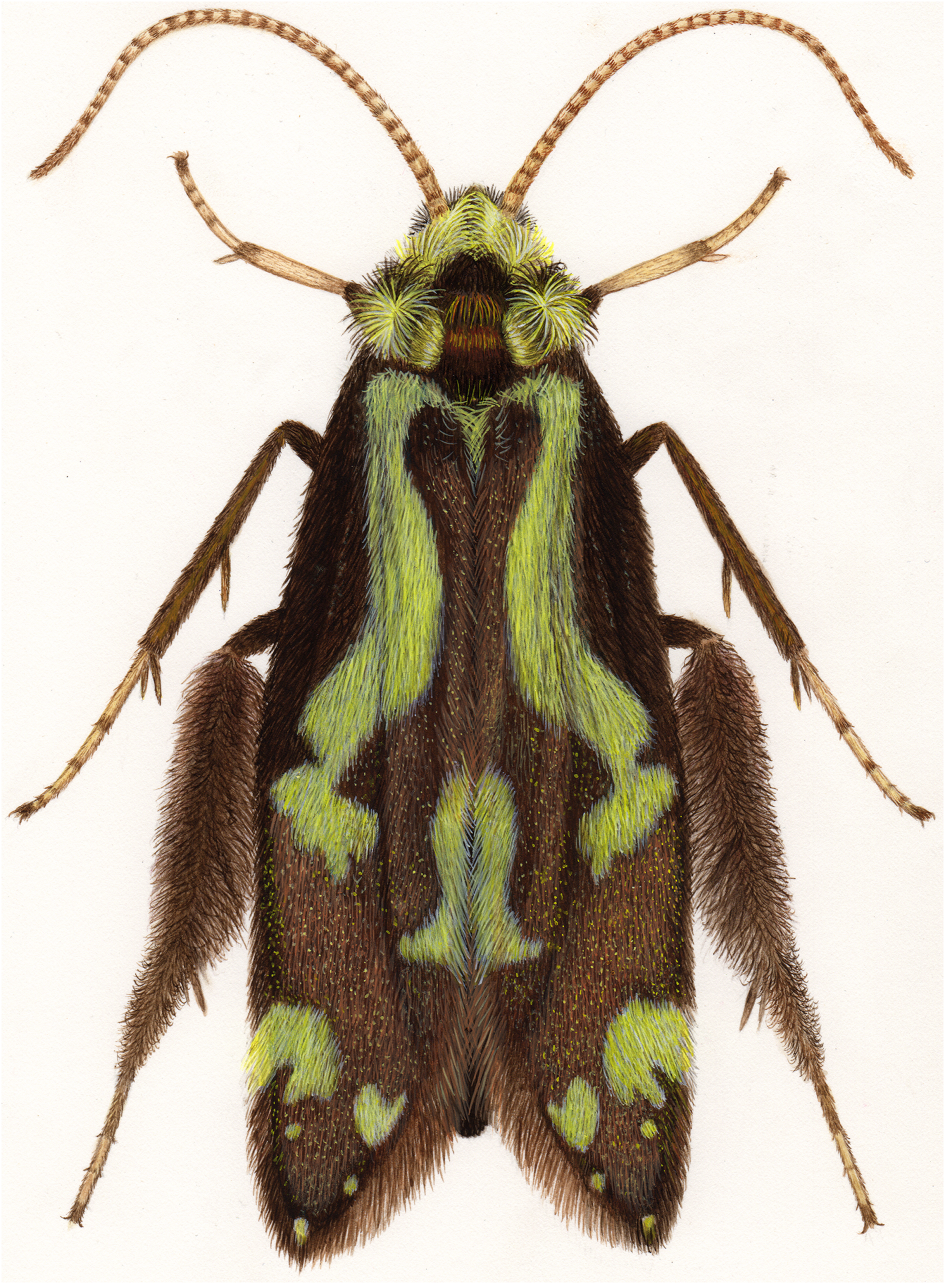
*Ascotrichia frontalis* Flint, 1983. Full habitus. Artwork credit: Julie Martinez.

Like other members of Hydroptilidae, *Ascotrichia* adults are minute, though they do represent some of the larger species in the family by reaching a length of 5.3 mm. Adults bear patches of bright green setae on their forewings, often observed as a faded green or yellow colour in older, pinned specimens (Fig. 1). Females have not been formally described or illustrated for any of the *Ascotrichia* species, but have been associated for some species due to locality and collection data that matches that of the holotype specimen. Larvae have not been associated for any species.

In 2011, Oláh and Johanson divided Leucotrichiinae into two generic clusters: the *Leucotrichia* genus cluster, which included *Abtrichia*, *Acostatrichia*, *Anchitrichia*, *Ascotrichia*, *Betrichia*, *Ceratotrichia*, *Costatrichia*, *Leucotrichia*, and *Zumatrichia*, and the *Celaenotrichia* genus cluster, which included *Alisotrichia*, *Byrsopteryx*, *Celaenotrichia*, *Cerasmatrichia*, *Mejicanotrichia*, and *Scelobotrichia*. The *Leucotrichia* genus cluster can be distinguished from the *Celaenotrichia* genus cluster by a modified spur formula and the typical leucotrichiine phallic median complex originally described in 1970 by Flint (Oláh & Johanson, 2011).

In the most recent phylogenetic analysis of Leucotrichiinae, *Ascotrichia* was recovered as monophyletic based on both morphological and molecular datasets (Santos, Nessimian & Takiya, 2016). *Ascotrichia* grouped with other genera from the *Leucotrichia* genus cluster (Oláh & Johanson, 2011) and was placed in the tribe Leucotrichiini Flint, 1970 sensu n., as modified by Santos, Nessimian & Takiya (2016). Analyses performed in this work suggest that *Ascotrichia* may be most closely related to the genus *Costatrichia* Mosely, 1937.

The genera traditionally recognised as members of the subfamily Leucotrichiinae have historically been difficult to separate (Flint, 1970; Marshall, 1979). Further work is needed to delimit the genera and properly assess their taxonomic and phylogenetic status. This review of *Ascotrichia* represents the first comprehensive work on the genus since its original description in 1983. All three previously known *Ascotrichia* species are re-described and illustrated, along with three new species, bringing the total number of species to six.

## Materials and Methods

### Specimen preparation and observation

To observe structural features of the male genitalia, soft tissues were cleared following procedures explained in detail by Blahnik, Holzenthal & Prather (2007). Abdomens, including genitalia, were removed from specimens using microscissors and placed individually in carefully labelled Pyrex^®^ test tubes (10 × 75 mm), each containing two to three ml of 85% lactic acid. Test tubes were then heated in a Fischer Scientific dry bath incubator at approximately 120 °C for 30–35 min. At the end of this time, abdomens were carefully removed from the test tubes and rinsed in ethanol to gently flush away any remaining lactic acid. For some specimens, the head was also removed and cleared to more easily observe any possible modifications or eversible structures that may have been obscured by dense setae. Specimens were then examined following the methods outlined in Thomson & Holzenthal (2015). Specifically, cleared genitalia were placed in a standard glass microscope depression slide (1.5 cm diameter × 3 mm deep well) with glycerin and glass microbeads (average diameter 0.5 mm). The glass microbeads held the genitalia in place and allowed structures to be viewed in precise lateral, dorsal, and ventral positions. Genitalia were examined with an Olympus BX43 compound microscope at 250–500 × magnification. Due to their small size and reduced venation, the wings of Hydroptilidae do not provide reliable taxonomic characters (Marshall, 1979; Schmid, 1998). For these reasons, forewing length only has been provided in species descriptions.

### Illustrations and descriptions

Specimen illustration and descriptions also followed the methods previously outlined in Thomson & Holzenthal (2015). Structures were traced in pencil with the use of an Olympus drawing attachment (model U-DA) mounted on the microscope. Pencil sketches were then scanned (Fujitsu ScanSnap S1500M scanner), edited in Adobe Photoshop (v.12.1; Adobe Systems Inc., San Jose, CA, USA), and used as a template in Adobe Illustrator (v.15.1.0, Adobe Systems Inc., San Jose, CA, USA) to be digitally inked. Electronic “drawing” was completed with the aid of a graphics tablet (Bamboo Pen; Wacom Company, Limited, Kazo, Japan). Species descriptions were constructed using the programme DELTA (Dallwitz, Paine & Zurcher, 1999), which uses a species × character state data matrix to produce natural-language descriptions and promote consistency in descriptive taxonomy.

Description of female specimens has been deferred for several reasons. Since female specimens were not available for all the species addressed in this study, comprehensive female descriptions for each species were not possible. Of the females examined, no noticeable or informative differences were observed. While some female specimens were collected from the same locality and date as males, there is still some uncertainty of association. For these reasons, species descriptions reflect observation of male specimens only. Females, when available, were included in “Material examined” for the purpose of establishing a record of occurrence and because presumptive association may prove useful for future studies.

### Morphological terminology

**Figure 2:**
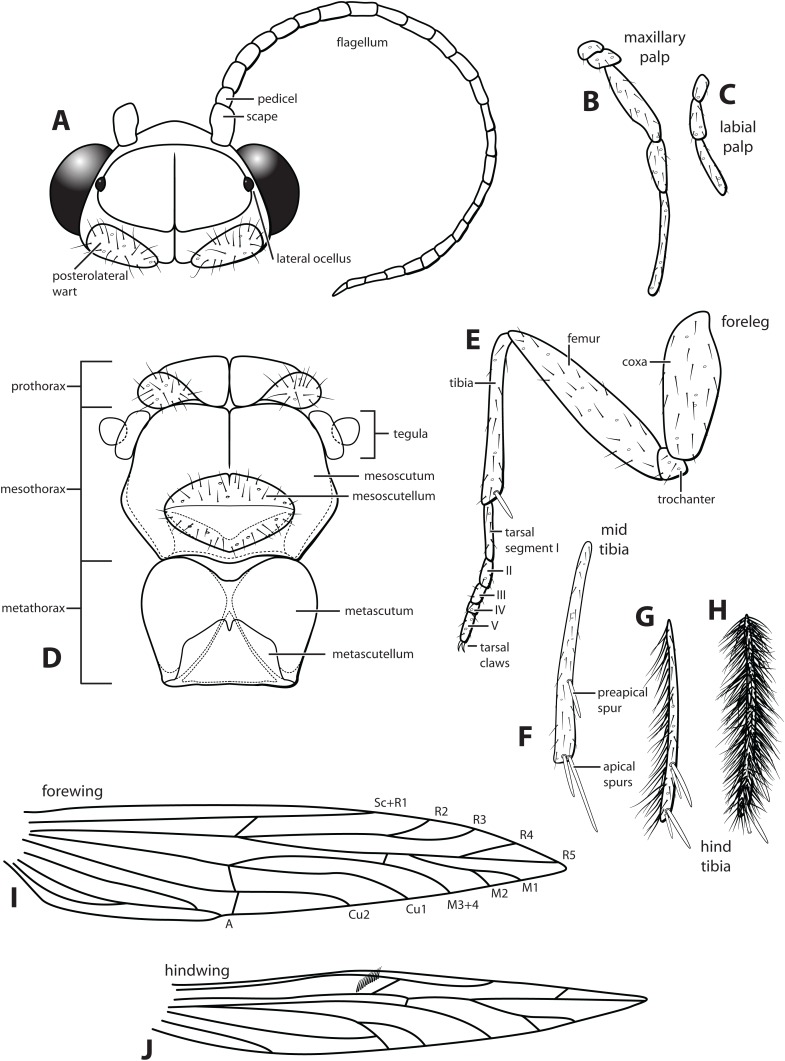
*Ascotrichia frontalis* Flint, 1983. (A) Head and antennae, dorsal. (B) Maxillary palp. (C) Labial palp. (D) Thorax, dorsal. (E) Foreleg with one tibial spur. (F) Mid tibia with three spurs. (G) Hind tibia without dense setae, with four spurs. (H) Hind tibia with dense setae. (I) Forewing. (J) Hindwing. Illustration credit: Robin Thomson.

**Figure 3:**
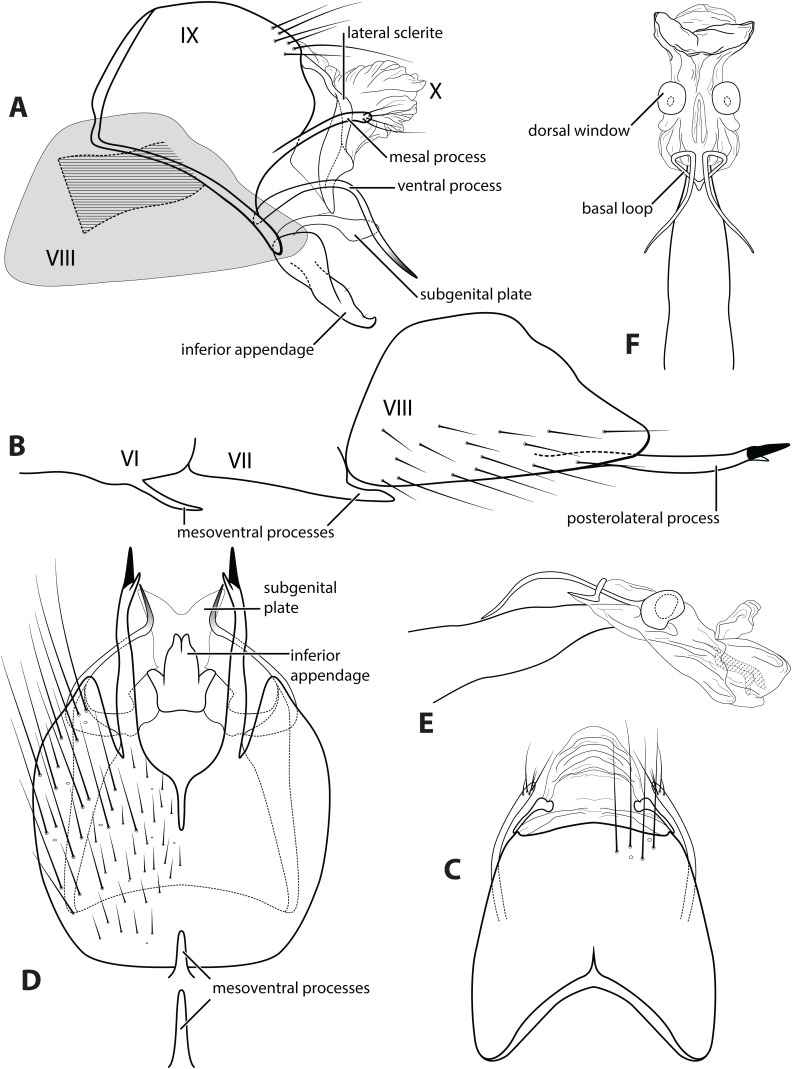
*Ascotrichia frontalis* Flint, 1983. Male genitalia: (A) Segments IX–X, segment VIII in grey, lateral (base of phallus crosshatched). (B) Segment VIII and mesoventral processes of segments VI–VII, lateral. (C) Segments IX–X, dorsal. (D) Segments VIII–IX and mesoventral processes of segments VI–VII, ventral. (E) Phallus, lateral. (F) Phallus, dorsal. Illustration credit: Robin Thomson.

Morphological terminology used for male genitalia was adapted from Flint (1983). For simplicity, paired structures are discussed in the singular. Terminology for specific structures is indicated in Figs. 2 and 3. The wing venation terminology of Fig. 2E follows the Comstock–Needham system as interpreted by Ross (1956) and Marshall (1979).

### Depositories

Types and material examined for this study are deposited at the Coleção Entomológica Professor José Alfredo Pinheiro Dutra, Departamento de Zoologia, Universidade Federal do Rio de Janeiro, Rio de Janeiro, Brazil (DZRJ); Museu de Zoologia, Universidade de São Paulo, São Paulo, Brazil (MZUSP); National Museum of Natural History, Washington, D.C., USA (NMNH); University of Minnesota Insect Collection, Saint Paul, Minnesota, USA (UMSP).

Specimen management followed the procedures outlined by Holzenthal & Andersen (2004). Each specimen deposited at the University of Minnesota Insect Collection (UMSP) that was examined during the study was affixed with a barcode label bearing a unique alphanumeric sequence beginning with the prefix UMSP. Holotype specimens being deposited at MZUSP were also affixed with a barcode label; this does not imply ownership by UMSP, but simply indicates that the specimen was databased at UMSP under that unique identification. Other specimens examined from other depositories were not given an additional UMSP barcode. Specimen-level taxonomic, locality, collection, and other information are stored in the University of Minnesota Insect Collection database using the software Specify 6.7.02 (Specify Collections Consortium, 2018).

### New species names

The electronic version of this article in portable document form will represent a published work according to the International Commission on Zoological Nomenclature (ICZN), and hence the new names contained in the electronic version are effectively published under that Code from the electronic edition alone. This published work and the nomenclatural acts it contains have been registered in ZooBank, the online registration system for the ICZN. The ZooBank Life Science Identifiers (LSIDS) can be resolved and the associated information viewed through any standard web browser by appending the LSID to the prefix http://zoobank.org/. The LSID for this publication is: urn:lsid:zoobank.org:pub:F9DD3403-78C8-45E2-87C1-F485CD62F7AD. The online version of this work is archived and available from the following digital repositories: PeerJ, PubMed Central, and CLOCKSS.

### Systematics

#### Generic description

**Genus *Ascotrichia*Flint, 1983**

*Ascotrichia* Flint, 1983:35 [Type species: *A. frontalis* Flint, 1983, original designation]. —Oláh & Johanson, 2011:152 [placement in *Leucotrichia* genus cluster]. —Santos, Nessimian & Takiya, 2016:11 [Leucotrichiinae phylogenetics]. —Holzenthal & Calor, 2017:202 [catalog].

**Diagnosis.** The original generic description suggested that *Ascotrichia* may be most similar to *Abtrichia*, but can be distinguished by the lack of extremely modified basal antennal segments present in *Abtrichia* species (Flint, 1983). The most recent phylogenetic analysis suggested that *Ascotrichia* may be most similar to *Costatrichia* (Santos, Nessimian & Takiya, 2016); the two genera can be easily distinguished by the number of ocelli present, *Ascotrichia* males having two and *Costatrichia* males having three.

**Description.** *Male*. Length of forewing ca. 3.0–5.3 mm. Wings unmodified, lacking a pouch, bulla, or patches of scales; forewing broad basally, acute apically; hind wing narrow, more acute than forewing, with row of hooked setae basal to cross vein *r* (Fig. 2E), edges with long setal fringe along posterior margin. Head with two ocelli, bearing setae and pair of setiferous posterolateral warts; antennae generally simple and unmodified, all flagellomeres of similar size and shape (Fig. 2A); observations of cleared heads did not reveal modifications as outlined in the original generic description. Maxillary palps with five segments; labial palps with three segments (Fig. 2B). Hind tibia sometimes with fringe of long setae down segment length, sometimes densely setose and resembling a bottlebrush; tibial spur count 1, 3, 4 (Fig. 2D). Mesoscutellum with transverse suture (Fig. 2C). Metascutellum pentagonal (Fig. 2C). *Genitalia*. Abdominal sternum VI with single mesoventral process (Figs. 3B and 3D). Abdominal sternum VII with single mesoventral process (Figs. 3B and 3D). Sternum VIII produced posteroventrally beneath segment IX (Fig. 3A), with simple, slender posterolateral process and posteromesal division. Segment IX open ventrally, sternum not developed (Fig. 3D), posterolateral margin with mesal and ventral processes; mesal process digitate and apically setose; ventral process long, slender, sometimes branched (Fig. 3A). Tergum X with heavily sclerotized lateral plates and membranous apex (Fig. 3A). Subgenital plate connected basally to base of inferior appendage, extending distally (Figs. 3A and 3D). Inferior appendage simple, elongate, sometimes fused mesoventrally (Figs. 3A and 3D). Phallus tubular basally, constricted at midlength with median complex bearing basal loop and pair of spherical “windows” (Figs. 3E and 3F); apex membranous, sac-like.

### Species descriptions

***Ascotrichia frontalis* Flint, 1983, type species**
Figs. 1–3

***frontalis*** Flint, 1983:36 [Type locality: Paraguay, Dpto. Alto Paraná, Salto del Monday, near Puerto Presidente Franco; NMNH; male]. —Angrisano, 1995:505 [distribution]. —Angrisano, 1999:31 [checklist]. —Paprocki, Holzenthal & Blahnik, 2004:10 [checklist]. —Dumas et al., 2009:366 [distribution]. —Oláh & Johanson, 2011:157 [distribution]. —Paprocki & França, 2014:41 [checklist]. —Santos, Nessimian & Takiya, 2016:465 [adult photograph].

**Diagnosis.** *Ascotrichia frontalis* is similar to *A. hystricosa* sp. n.; both species have subapical spines present on the posterolateral process of sternum VIII. *Ascotrichia frontalis* can be distinguished by the absence of subapical spines on the ventral process of segment IX (Figs. 3A and 3D), which are present in *A. hystricosa*.

**Description.** *Male*. Length of forewing 4.0–5.3 mm (*n* = 64). Dorsum of head brown with light yellow setae; thorax brown with brown setae dorsally, yellow laterally, brown ventrally; leg segments brown with brown setae; some specimens observed with densely setose, bottlebrush hind tibia (Fig. 1). Forewings covered with fine light yellow setae on basal 1/3, distal 2/3 with fine brown setae and patches of varying size of light yellow setae (Fig. 1). *Genitalia*. Abdominal sternum VI mesoventral process elongate, slender. Abdominal sternum VII mesoventral process digitate. Sternum VIII with posterior margin produced in broadly rounded apex; in ventral view posterior margin concave, with narrow mesal emargination; posterolateral process long, slender, with dark, pointed, stout apical spine and small preapical spine on inner face. Segment IX anterolateral margin convex, posterolateral margin slightly produced mesally; dorsally, anterior margin concave, posterior margin very shallowly convex (almost straight); mesal process slender, digitate, apically setose; ventral process arched mesally with apex extending ventrad, apex pointed and darkly sclerotized; in ventral view, process extends mesally with apex bending distally at a near-right angle. Tergum X with lateral sclerite with crenulate posterior margin; membranous apex suborbicular. Subgenital plate slender in lateral view, with ventral production subapically; broad ventrally with subtriangular apical emargination. Inferior appendage rounded basally and with slight apical hook, dorsal surface sparsely setose; ventrally fused, with small apical incision. Phallus apex with pair of membranous dorsal lobes.

**Material examined.** *Paratypes:* **BRAZIL: Espírito Santo:** Fazendo Santa Clara, 15 km SE Santa Teresa, 22.iv.1977, C.M. & O.S. Flint, Jr., one male (NMNH); **PARAGUAY: Alto Paraná:** Salto del Monday (near Puerto Presidente Franco), 26.xi.1973, O.S. Flint, Jr., three males, one female (NMNH). *Nontypes:* **BRAZIL: Minas Gerais:** Serra do Cipó, Rio Cipó in Cardeal Mota, 19°21.011S, 43°38.171′W, el. 720 m, 11.iii.1996, Holzenthal, Rochetti, and Oliveira, six males, 35 females (UMSP); Serra do Cipó, Rio Cipó in Cardeal Mota (Cach. Baixo), 19°20.553′S, 43°38.531′W, el. 750 m, 10-15.ii.1998, Holzenthal, Paprocki, and Huisman, one male, three females (UMSP); Serra do Cipó, Rio Cipó in Cardeal Mota (Cach. Grande), 19°21.011′S, 43°38.171′W, el. 720 m, 5.ii.1998, Holzenthal & Paprocki, two males, 10 females (UMSP); Rio Tanque, ca. 12 km (rd) from Ipoema, 19°32.208′S, 43°26.878′Wm el. 750 m, 16.v.1998, Holzenthal & Paprocki, 14 males, 13 females (UMSP); Santana do Riacho, Rio Cipó, Cach. Tomé, 19°20′48″S, 43°38′16″W, 21.iv.2010, el. 779 m, light trap, A.P.M. Santos, L.L. Dumas, D.M. Tikaya, one male (in alcohol) (DZRJ); **Rio de Janeiro:** Encontro dos Rios (Macaé/Bonito), six km S Lumiar, 22°23.484′S, 42°18.698′W, el. 600 m, 10.iii.2002, Holzenthal, Blahnik, Paprocki, Prather, 28 males, 13 females (UMSP); **São Paulo:** Salesópolis, Res. Biol. Boraceia, el. 891 m, 23°33′55″S, 45°52′43″W, 28.i.2011, light trap, A.P.M. Santos, D.M. Takiya, L.L. Dumas, J.L. Nessimian, seven males, three females (in alcohol) (DZRJ); **Espirito Santo:** Santa Leopoldina, Rio da Prata, el. 431 m, 20°02′55″S, 40°31′57″W, 3.iv.2011, light trap, L.L. Dumas, G.A. Jardim, J.L. Nessimian, one male, four females (in alcohol) (DZRJ).

**Etymology.** *Frontis*, Latin for “brow, forehead,” likely referring to the large seta-filled pocket in the head observed by Flint.

**Figure 4:**
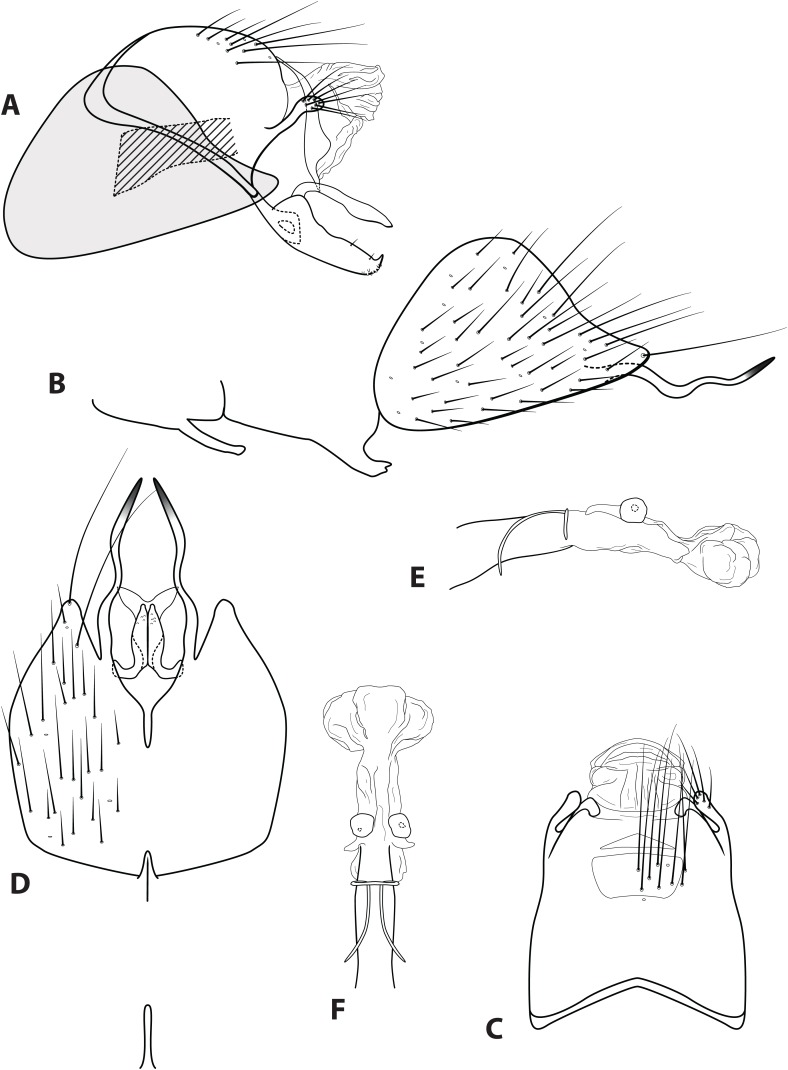
*Ascotrichia adirecta* sp. n. (UMSP000030826). Male genitalia: (A) Segments IX–X, segment VIII in grey, lateral (base of phallus crosshatched). (B) Segment VIII and mesoventral processes of segments VI–VII, lateral. (C) Segments IX–X, dorsal. (D) Segments VIII–IX and mesoventral processes of segments VI–VII, ventral. (E) Phallus, lateral. (F) Phallus, dorsal. Illustration credit: Robin Thomson.

***Ascotrichia adirecta* Thomson, sp. n.**
LSID urn:lsid:zoobank.org:act:21426ACD-AC4D-4C12-BB6C-2BE7218A27DC
Fig. 4

**Diagnosis.** This species is unique within the genus due to the absence of the ventral process on segment IX, which is present on all the other *Ascotrichia* species (Fig. 4A).

**Description.** *Male*. Length of forewing 4.2–4.7 mm (*n* = 6). Dorsum of head with yellow setae and small patch of dark brown setae basally; thorax brown with dark brown setae dorsally, light yellow laterally; leg segments brown with brown setae; all specimens observed with densely setose, bottlebrush hind tibia. Forewings covered with fine dark brown setae, with patches of light yellow setae. *Genitalia*. Abdominal sternum VI mesoventral process slender, apex truncate, ventrally digitate. Abdominal sternum VII mesoventral process short, stout, apex with small acute emargination, ventrally apex pointed. Sternum VIII subtriangular, with posterior margin produced ventrally; in ventral view posterior margin concave, with narrow mesal emargination; posterolateral process slender, sinuous, extending posteriad, apex darkly sclerotized. Segment IX anterolateral margin convex, posterolateral margin convex; dorsally, anterior margin concave, posterior margin very shallowly convex (almost straight); mesal process apex slightly bulbous, apically setose; ventral process absent. Tergum X with lateral sclerite with crenulate posterior margin; membranous apex amorphous. Subgenital plate slender, with slight subapicoventral hump, apex pointed; in ventral view apex flared out with broad emargination. Inferior appendage with dorsobasal hump, apical 1/3 of dorsal surface sparsely setose, subapicoventral surface rugose, apex with small dorsal hook; ventrally with apex digitate. Phallus apex amorphous, bulbous in dorsal view.

*Holotype male:* **BRAZIL: Minas Gerais:** confluence Rio Peixe & Rio Preto do Itambé, 19°17.525′S, 43°15.547′W, el. 500 m, 4.ii.1998, Holzenthal & Paprocki (UMSP000030826) (MZUSP). *Paratype:* same data as holotype, one male (UMSP); **BRAZIL: Bahia:** three km de Una, Res. Biol. de Una, 15°17′29″S, 36°06′09″, 14.vi.2014, W.R.M. Souza, A.P.M. Santos, D.M. Takiya, four males (DZRJ). *Other material examined:* same data as holotype, one female (UMSP).

**Etymology.** A, Latin for “not;” *directus*, Latin for “set straight, arrange in a straight line,” referring to the sinuous nature of the posterolateral process on sternum VIII.

**Figure 5:**
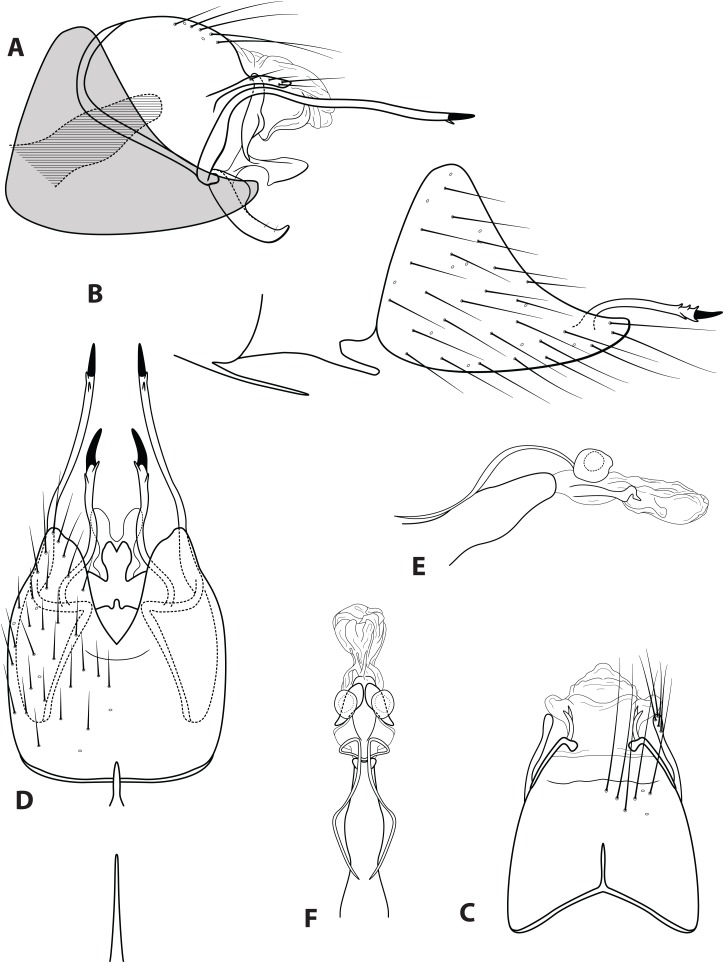
*Ascotrichia hystricosa* sp. n. (UMSP000021862). Male genitalia: (A) Segments IX–X, segment VIII in grey, lateral (base of phallus crosshatched). (B) Segment VIII and mesoventral processes of segments VI–VII, lateral. (C) Segments IX–X, dorsal. (D) Segments VIII–IX and mesoventral processes of segments VI–VII, ventral. (E) Phallus, lateral. (F) Phallus, dorsal. Illustration credit: Robin Thomson.

***Ascotrichia hystricosa* Thomson, sp. n.**
LSID urn:lsid:zoobank.org:act:085DBA50-F1F0-4925-8A81-ABC3C6F05669
Fig. 5

**Diagnosis.** *Ascotrichia hystricosa* is similar to *A. frontalis*; both species have subapical spines present on the posterolateral process of sternum VIII. *Ascotrichia hystricosa* can be distinguished by the presence of subapical spines present also on the ventral process of segment IX (Figs. 5A and 5D), which are absent in *A. frontalis*.

**Description.** *Male*. Length of forewing 3.5–4.6 mm (*n* = 18). Dorsum of head with yellow setae and small patch of dark brown setae basally; thorax brown with dark brown setae dorsally, light yellow laterally; leg segments brown with brown setae; no specimens observed with densely setose, bottlebrush hind tibia. Forewings covered with fine light yellow setae on apical 1/3, distal 2/3 with fine brown setae and patches of varying size of light yellow setae. *Genitalia*. Abdominal sternum VI mesoventral process extremely slender, apex acuminate. Abdominal sternum VII mesoventral process digitate. Sternum VIII subtriangular, with posterior margin produced ventrally; in ventral view posterior margin acutely concave; posterolateral process reaching dorsad from base, then bending at right angle and extending posteriad, apex with small subapical spines and single large dark apical spine. Segment IX anterolateral margin convex, posterolateral margin convex; dorsally, anterior margin concave, posterior margin straight; mesal process slender, digitate, apically setose; ventral process elongate, slender, broadest basally, basal 1/3 extending dorsad and apical 2/3 extending posteriad to create an approximately 90° angle, apex darkly sclerotized, in ventral view apex with short subapical spines. Tergum X with lateral sclerite with crenulate posterior margin; membranous apex amorphous. Subgenital plate with narrow mesal constriction in lateral view, with ventral production subapically; ventrally with rounded emargination creating two rounded apical lobes. Inferior appendage rounded basally, apical 1/3 of dorsal surface setose, apex curled slightly dorsad; ventrally fused, apex with small acute emargination. Phallus apex amorphous.

*Holotype male:* **BRAZIL: Minas Gerais:** Serra do Cipó, Rio Cipó in Cardeal Mota, 19°21.011′S, 43°38.171′W, el. 720 m, 11.iii.1996, Holzenthal, Rochetti, Oliveira (UMSP000021862) (MZUSP). *Paratypes:* **BRAZIL: Minais Gerais:** Rio Paraúna, three km S Santana do Riacho, 19°10.986′S, 43°43.485′W, el. 650 m, 16.ii.1998, Holzenthal & Paprocki, two males (UMSP); Rio Paraúna, three km S Santana do Riacho, 19°10.986′S, 43°43.485′W, el. 650 m, 17.v.1998, Holzenthal & Paprocki, four males (UMSP); Rio Tanque, ca. 12 km (rd) from Ipoema, 19°32.208′S, 43°26.878′W, el. 750 m, 16.v.1998, Holzenthal & Paprocki, four males (UMSP); **Goias:** Mineiros, Rio Vendinha, 17°35′21″S, 52°35′59″W, 23.ii.2012, el. 633 m, light trap, L. Sgarbi, A.P.M. Santos, E. Raimundi, three males (in alcohol) (DZRJ); Portelândia, riacho, 17°28′30″S, 52°41′05″W, 20.ii.2012, el. 819 m, light trap, L. Sgarbi, A.P.M. Santos, E. Raimundi, four males (in alcohol) (DZRJ). *Other material examined:* same data as holotype, one female (UMSP); Rio Paraúna, three km S Santana do Riacho, 19°10.986′S, 43°43.485′W, el. 650 m, 16.ii.1998, Holzenthal & Paprocki, five females (UMSP); Rio Paraúna, three km S Santana do Riacho, 19°10.986′S, 43°43.485′W, el. 650 m, 17.v.1998, Holzenthal & Paprocki, 15 females (UMSP).

**Etymology.** *Hystricosus*, Latin for “prickly, thorny,” referring to apical spines of both the posterolateral process on sternum VIII and the ventral process of segment IX.

**Figure 6:**
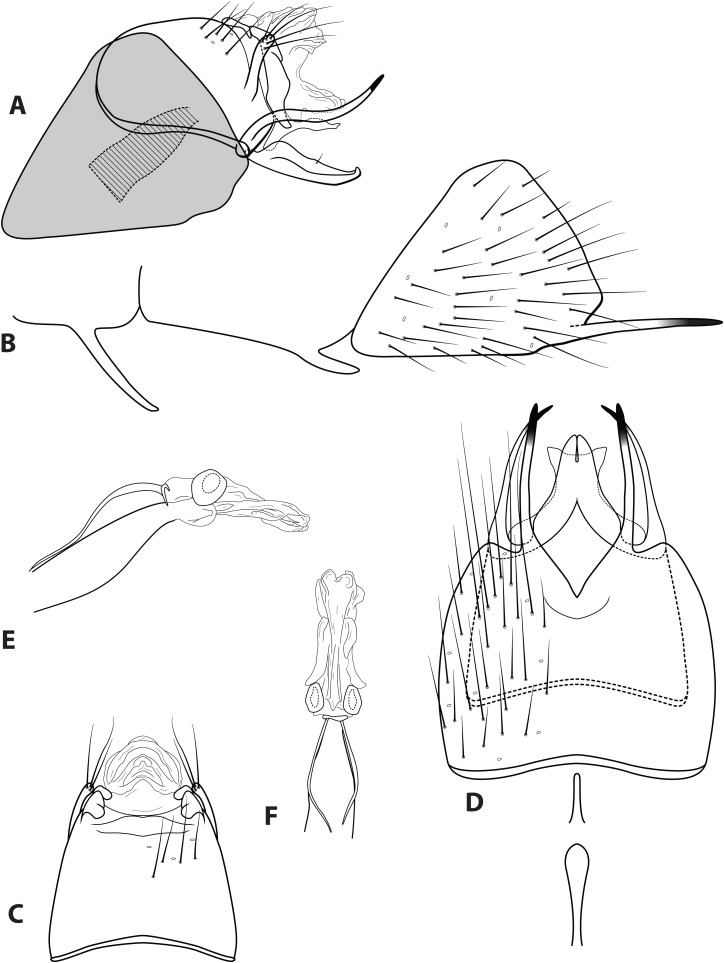
*Ascotrichia simoma* sp. n. (UMSP000030569). Male genitalia: (A) Segments IX–X, segment VIII in grey, lateral (base of phallus crosshatched). (B) Segment VIII and mesoventral processes of segments VI–VII, lateral. (C) Segments IX–X, dorsal. (D) Segments VIII–IX and mesoventral processes of segments VI–VII, ventral. (E) Phallus, lateral. (F) Phallus, dorsal. Illustration credit: Robin Thomson.

***Ascotrichia simoma* Thomson, sp. n.**
LSID urn:lsid:zoobank.org:act:2558BC1D-1469-459F-82A5-5045B0EE7099
Fig. 6

**Diagnosis.** *Ascotrichia simoma* sp. n. is unique within the genus due to the apex of the ventral process on segment IX extending dorsally (Fig. 6A). In all other species in the genus, the apex of this process extends either distally or ventrally.

**Description.** *Male*. Length of forewing 4.8–5.1 mm (*n* = 10). Dorsum of head with dark brown setae, some yellow setae apically near antennae; thorax brown with dark brown setae dorsally, light yellow laterally; leg segments brown with brown setae; no specimens observed with densely setose, bottlebrush hind tibia. Forewings covered with fine light yellow setae on apical 1/3, distal 2/3 with fine brown setae and patches of varying size of light yellow setae. *Genitalia*. Abdominal sternum VI mesoventral process elongate, slender, in ventral view apex slightly bulbous. Abdominal sternum VII mesoventral process short, knife-like, in ventral view slender and digitate. Sternum VIII with posterior margin produced in rounded apex; in ventral view posterior margin acutely concave; posterolateral process slender, extending straight posteriad, apical 1/4 darkly sclerotized. Segment IX anterolateral margin convex, posterolateral margin slightly produced mesally; dorsally, anterior margin shallowly concave, posterior margin shallowly concave; mesal process digitate, extending dorsad, apically setose; ventral process slender, slightly sinuous, apex extending dorsad, apex darkly sclerotized, in ventral view apex slightly curved inward. Tergum X with lateral sclerite divided into simple dorsal sclerite and larger ventral sclerite with shallowly crenulate posterior margin; membranous apex divided into amorphous dorsal lobe and amorphous ventral lobe. Subgenital plate broadest basally, with crenulate dorsal margin, apex truncate with small apicodorsal production; in ventral view with apex slightly flaring outwards and shallowly concave emargination. Inferior appendage with basal hump, single mesodorsal seta, apex with small dorsal hook; ventrally fused, with narrow emargination reaching 1/4 the total length creating two digitate lobes, lobe apices touching. Phallus apex amorphous, with possible internal structures obscured from view.

*Holotype male:* **BRAZIL: São Paulo:** Estação Biológica Boraceia, Rio Guaratuba, 23°40.039′S, 45°53.759′W, el. 775 m, 17.iv.1998, Holzenthal, Melo, Froehlich (UMSP000030569) (MZUSP). *Paratypes:* same data as holotype, seven males (UMSP); **Minas Gerais:** Córrego da Serra de Ouro Fino, Vale do Tropeiro, 20°12.371′S, 43°38.581′W, el. 1,000 m, 08.x.2000, Paprocki, Salgado, Isaac, two males (UMSP). *Other material examined:* Same data as holotype, four females (UMSP); **Minas Gerais:** Córrego da Serra de Ouro Fino, Vale do Tropeiro, 20°12.371′S, 43°38.581′W, el. 1,000 m, 08.x.2000, Paprocki, Salgado, Isaac, eight females (UMSP).

**Etymology.** *Simoma*, Greek for “anything turned up”, referring to the apex of the ventral process on segment IX extending dorsally.

**Figure 7:**
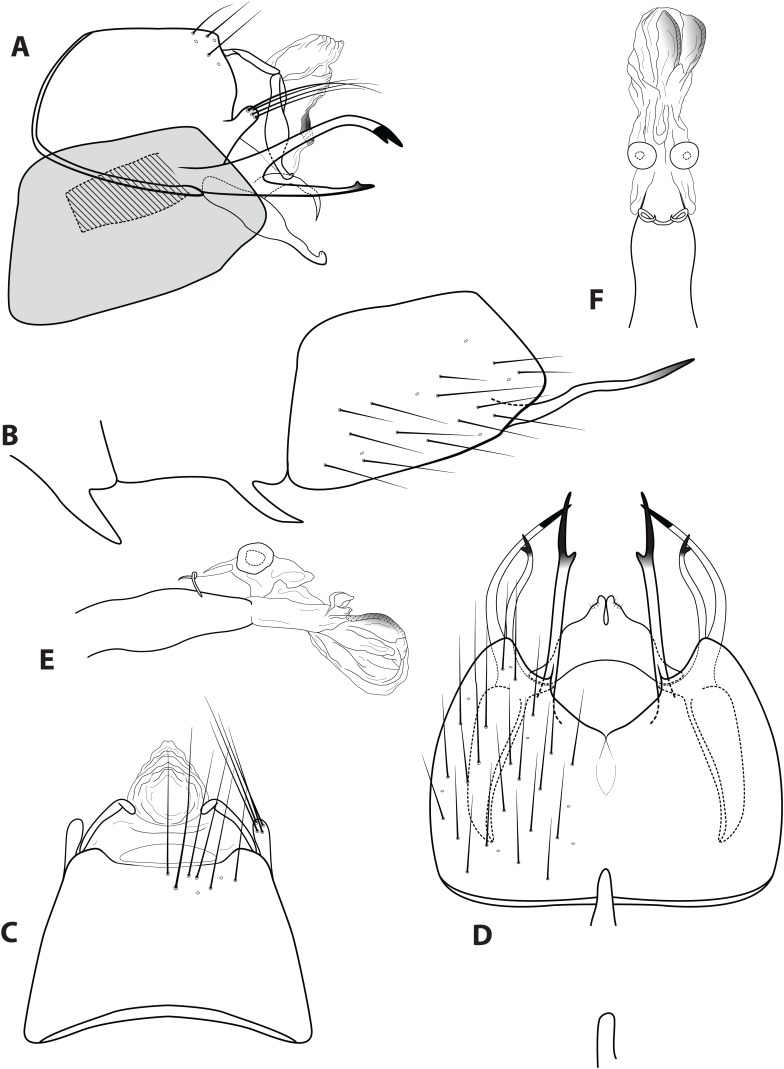
*Ascotrichia spangleri* Oláh & Flint, 2012. Male genitalia: (A) Segments IX–X, segment VIII in grey, lateral (base of phallus crosshatched). (B) Segment VIII and mesoventral processes of segments VI–VII, lateral. (C) Segments IX–X, dorsal. (D) Segments VIII–IX and mesoventral processes of segments VI–VII, ventral. (E) Phallus, lateral. (F) Phallus, dorsal. Illustration credit: Robin Thomson.

***Ascotrichia spangleri* Oláh and Flint, 2012**
Fig. 7

***spangleri*** Oláh & Flint, 2012:167 [Type locality: Venezuela, Amazonas Federal Territory, Puerto Ayacucho (40 km South), El Tobogan, Cano Coromoto; NMNH; male].

**Diagnosis.** *Ascotrichia spangleri* is most similar to *A. surinamensis*, due to the branched ventral process on segment IX, which is simple in all other species in the genus. *Ascotrichia spangleri* can be distinguished by the absence of three strong spines on the ventral branch of the process (Fig. 7A), which are present in *A. surinamensis*.

**Description.** *Male*. Length of forewing 3.0–4.7 mm (*n* = 6). Dorsum of head with light yellow setae apically and dark brown setae basally; thorax brown with brown setae; leg segments brown with brown setae; no specimens observed with densely setose, bottlebrush hind tibia. Forewings covered with fine dark brown setae mottled with light yellow–green setae on apical 1/3, distal 2/3 dark brown with small patches of light yellow setae. *Genitalia*. Abdominal sternum VI mesoventral process pointed laterally, digitate ventrally. Abdominal sternum VII mesoventral process pointed, in ventral view digitate. Sternum VIII subquadrate with convex posterior margin; in ventral view posterior margin concave, with oval emargination mesally; posterolateral process slightly sinuous in lateral view, apex darkly sclerotized, small subapical spine apparent in ventral view. Segment IX anterolateral margin convex, posterolateral margin straight; dorsally, anterior margin shallowly concave, posterior margin with broad, straight mesal emargination; mesal process short, stout, apically setose; ventral process branched, both branches long and slender; dorsal branch arched, apex darkly sclerotized and bifid in lateral view with ventral point reaching about midlength of dorsal point; ventral branch straight, extending dorsad, apex darkly sclerotized and with small, pointed subapical projection. Tergum X with lateral sclerite divided into simple dorsal sclerite and larger ventral sclerite with shallowly crenulate posterior margin; membranous apex amorphous. Subgenital plate arched, with pointed apex extending ventrad; obscured by inferior appendage in ventral view. Inferior appendage subquadrate basally, broadest mesally, digitate apex hooked; ventrally with shallowly crenulate outer margin, apex sparsely setose and with narrow emargination. Phallus apex amorphous, with possible internal structures obscured from view.

**Material examined.** *Paratypes:* **VENEZUELA: Amazonas Federal Territory [Amazonas State]:** Puerto Ayacucho (40 km South), El Tobogán, 14.xi.1987, coll'n. #1, P.J. Spangler & R.A. Faitoute, collected at blacklight, three males (NMNH); Amazonas Federal Territory, Puerto Ayacucho (40 km S) El Tobogán, Caño Coromoto, 18.i.1989, blacklight, upper shelter, collected by P.J. Spangler, R.A. Faitoute, and C.B. Barr, three males (NMNH).

**Etymology.** Presumably named after Paul Spangler, one of the collectors of the holotype specimen.

***Ascotrichia surinamensis*** (Flint, 1974)
Fig. 8

**Figure 8:**
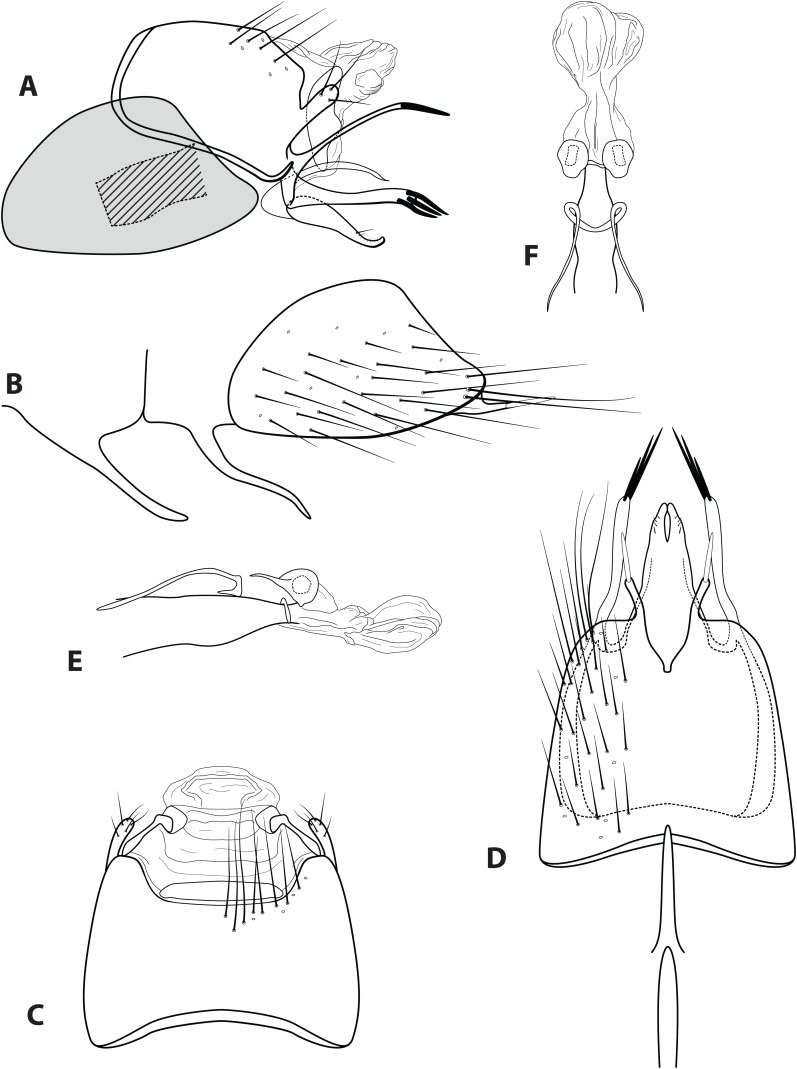
*Ascotrichia surinamensis* (Flint, 1974). Male genitalia: (A) Segments IX–X, segment VIII in grey, lateral (base of phallus crosshatched). (B) Segment VIII and mesoventral processes of segments VI–VII, lateral. (C) Segments IX–X, dorsal. (D) Segments VIII–IX and mesoventral processes of segments VI–VII, ventral. (E) Phallus, lateral. (F) Phallus, dorsal. Illustration credit: Robin Thomson.

***surinamensis*** (Flint), 1974:57 [Type locality: Suriname, Nickerie River, Blanche Marie; RNH; males; in *Betrichia*]. —Flint, 1983:36 [to *Ascotrichia*]. —Oláh & Johanson, 2011:157 [distribution].

**Diagnosis.** *Ascotrichia surinamensis* is most similar to *A. spangleri*, due to the branched ventral process on segment IX, which is simple in all other species in the genus. *Ascotrichia surinamensis* can be distinguished by the presence of three strong spines on the ventral branch of the process (Fig. 8A), which are absent in *A. spangleri*.

**Description.** *Male*. Length of forewing 3.5–5.0 mm (*n* = 7). Dorsum of head with yellow setae and small patch of dark brown setae basally; thorax brown with dark brown setae dorsally, light yellow laterally; leg segments brown with brown setae; no specimens observed with densely setose, bottlebrush hind tibia. Forewings covered with fine dark brown setae, with large amorphous patch of fuscous yellow setae. *Genitalia*. Abdominal sternum VI mesoventral process elongate, slender. Abdominal sternum VII mesoventral process elongate, slender, slightly sinuous in lateral view. Sternum VIII with posterior margin produced in rounded apex; in ventral view posterior margin concave, with truncate emargination apex; posterolateral process short, slender, digitate, with stout dark apical spine. Segment IX anterolateral margin convex, posterolateral margin convex; dorsally, anterior margin shallowly concave, posterior margin with broad, straight mesal emargination; mesal process stout, digitate, apically setose; ventral process branched; dorsal branch elongate, very slender, apical 1/4 darkly sclerotized, apex acuminate; ventral branch narrowest mesally, apex digitate and with three dark, stout spines. Tergum X with lateral sclerite having crenulate posterior margin; membranous apex amorphous. Subgenital plate slender, arched, with large rounded base, apex acute; obscured by inferior appendage in ventral view. Inferior appendage rounded basally, broadest basally, tapering apically, apex with short setae dorsally; ventrally fused, elongate, apical 1/4 with narrow emargination. Phallus apex amorphous, bulbous in dorsal view.

**Material examined.** *Paratypes:* **SURINAME:** Tapanahoni River, Grandafoetoe, at light, 13.iii.1952, D.C. Geljskes, one male (NMNH); Blanche Marie, behind camp, 15.ii.1971, at light, D.C. Geljskes, three males (NMNH); **GUYANA:** Kanuku Mts., Moco Moco River, 3°18.2′N, 59°38.9′W, 29.iv.1995, O.S. Flint, Jr., one male (NMNH); Potaro R., Kaieteur Falls, 1350′, 5°10.5′N, 59°28.8′W, 21–23.viii.1997, O.S. Flint, Jr., two males (NMNH).

**Etymology.** Presumably named for the country of Suriname, where the type specimen was collected.

## Conclusions

This revision doubles the number of known species within the microcaddisfly genus *Ascotrichia* (Trichoptera, Hydroptilidae) from three to six. All three of the new species are known only from collections made in Brazil. This highlights the need for more taxonomic research focused on insect fauna in both the country of Brazil specifically, but also in South America in general. Both fieldwork and surveys of unidentified specimens in already-existing insect collections need to be conducted in order to establish a better picture of the insect diversity contained within South America.

## Supplemental Information

10.7717/peerj.7560/supp-1 Supplemental Information 1 Specimen information: specimen numbers, depositories, genus, and species.

## Additional Information and Declarations

### Data Availability

The following information was supplied regarding data availability: All specimens stored permanently at the University of Minnesota have been assigned a unique ID number; unique IDs have also been assigned to holotype specimens for new species described here, as they are specimens of particular value. The genus, species, specimen ID, depository location, and curator contact information for the collections are available in a Supplemental File.

### New Species Registration

The following information was supplied regarding the registration of a newly described species: Publication LSID: urn:lsid:zoobank.org:pub:F9DD3403-78C8-45E2-87C1-F485CD62F7AD *Ascotrichia adirecta* sp. n. LSID: urn:lsid:zoobank.org:act:21426ACD-AC4D-4C12-BB6C-2BE7218A27DC. A*scotrichia hystricosa* sp. n. LSID: urn:lsid:zoobank.org:act:085DBA50-F1F0-4925-8A81-ABC3C6F05669. *Ascotrichia simoma* sp. n. LSID: urn:lsid:zoobank.org:act:2558BC1D-1469-459F-82A5-5045B0EE7099.
